# Cartilage Defect Articulation With the Proximal Phalanx After Retrograde Intramedullary Screw Fixation of Metacarpal Fractures: A Cadaveric Study

**DOI:** 10.1016/j.jhsg.2025.100847

**Published:** 2025-10-14

**Authors:** Clayton Hui, Benjamin Watzig, Luke Nicholson, Joshua W. Hustedt

**Affiliations:** ∗ Department of Orthopedic Surgery, University of Arizona College of Medicine-Phoenix, Phoenix, AZ; † Department of Orthopedic Surgery, University of Southern California, Los Angeles, CA

**Keywords:** Bones of hand, Fracture, Metacarpal, Metacarpal phalangeal joint, Orthopedic

## Abstract

**Purpose:**

Retrograde intramedullary screw fixation for metacarpal fractures has become increasingly popular; however, the technique requires violation of the extensor mechanism at the metacarpophalangeal (MCP) joint and penetration of the metacarpal articular surface. Although prior studies have quantified the proportion of metacarpal articular surface involved in screw placement, none have evaluated how the resulting cartilage defect articulates with the proximal phalanx at the MCP joint as a potential source of altered wear patterns.

**Methods:**

In a cadaveric study, six specimens (24 metacarpals) underwent fixation of simulated metacarpal neck fractures with either a 3.6 or a 4.5 mm metacarpal-specific screw inserted via peritendinous or percutaneous approach. The location and percentage of the metacarpal head articular surface damage were measured. The MCP joint was then taken through a full arc of motion in all planes to map contact between the metacarpal defect and the proximal phalanx articular cartilage.

**Results:**

Retrograde screw placement resulted in an average of 8% metacarpal articular surface damage. The percutaneous approach produced a significantly more volar starting point compared with the peritendinous approach. Across the simulated arc of motion, 100% of the proximal phalanx articular cartilage came into contact with the cartilage defect on the metacarpal head.

**Conclusions:**

Retrograde metacarpal screw fixation produces a focal cartilage defect on the metacarpal head that tracks across the entire proximal phalanx articular surface during MCP joint motion.

**Clinical relevance:**

This focal damage articulating with the entire base of the phalanx may alter contact stress, potentially accelerating degradation and predisposing to MCP arthritis over time.

Retrograde intramedullary screw fixation for metacarpal fractures has gained popularity in recent years.^1^, ^2^, ^3^, ^4^, ^5^, ^6^ Short-term clinical studies report high union rates with good functional outcomes, and early comparative studies suggest that retrograde fixation may allow quicker return of function with less stiffness and adhesion than traditional fixation methods such as K-wires or plates and screws.^5^^,^^7^

Despite these advantages, retrograde screw placement requires violation of the extensor mechanism at the metacarpophalangeal (MCP) joint and penetration of the metacarpal head articular surface (Fig. 1). Both may have long-term consequences for MCP joint function. Ruchelsman et al^8^ reported extensor lag in 50% of patients following retrograde fixation, although most cases resolve over time, and del Piñal et al^9^ described two patients with permanent extensor lag >30 degrees in a series of 48 metacarpals. Of potentially greater concern is the necessity to breach the articular surface during screw placement. Eisenberg et al^10^ observed MCP arthritis in one of 86 metacarpal fracture fixations, likely related to articular surface damage related to screw placement.

**Figure 1:**
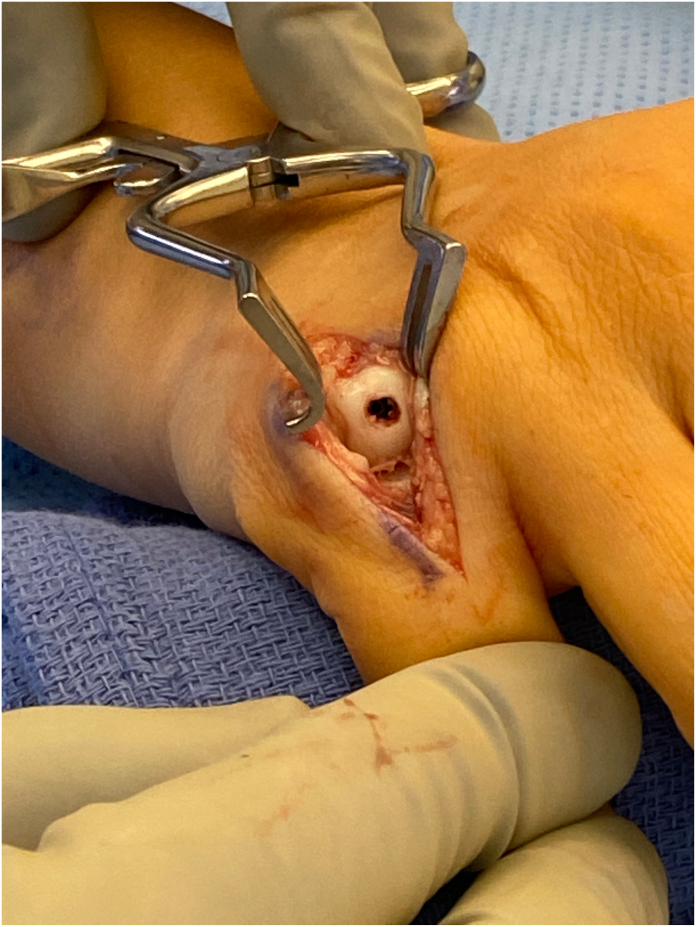
Representative photograph of the articular surface damage to the metacarpal head with retrograde screw fixation.

Articular surface violation and its potential association with the development of MCP arthritis remain a key concern with this technique. Multiple cadaveric studies have quantified the percentage of metacarpal head articular surface violation by retrograde screws, but these have focused exclusively on the metacarpal side.^9^^,^^11^^,^^12^ No prior study has examined the pattern or degree of articulation of the proximal phalanx surface in contact with the cartilage defect on the metacarpal head. Since cartilage degeneration results from the interaction between opposing joint surfaces, assessing only the metacarpal side may underestimate the true risk.

We hypothesize that quantifying the proportion of the proximal phalanx articular surface that contacts the damaged region of the metacarpal head during full MCP joint motion will provide a more clinically relevant indicator of long-term cartilage wear and arthritis risk. The present study was designed to map this functional wear pattern in a cadaveric model of retrograde screw fixation for metacarpal neck fractures.

## Materials and Methods

### Cadaveric fracture fixation model

**Figure 2:**
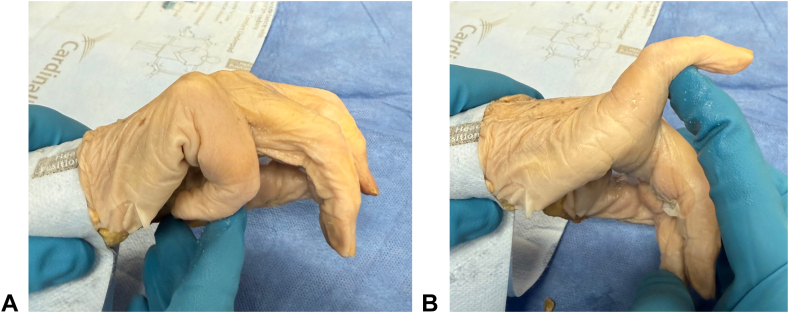
Representative photograph of the range of motion (ROM) of the MCP joint in the cadaveric specimens. **A** Maximum flexion. **B** Maximum extension of the MCP joint.

Six fresh-frozen cadaveric specimens (24 metacarpals) were used in this study. Baseline range of motion of the MCP joints for the index through little fingers was measured using a goniometer (Fig. 2). Metacarpal neck fractures were then simulated using an oscillating saw. Neck fractures were chosen over midshaft fractures because they are the most common type treated with intramedullary screws. Additionally, prior authors have suggested that a more volar starting point on the metacarpal head may be necessary to achieve proper reduction and avoid comminution in neck fractures.^5^^,^^13^^,^^14^ This volar starting point may increase articular surface damage at the MCP joint, making neck fractures an appropriate model to assess articular involvement in retrograde screw fixation.

### Surgical approach and fracture fixation

Three cadavers underwent fixation via an open paratendinous approach, and three via a percutaneous approach. Both methods were chosen to reflect recent trends favoring percutaneous fixation.^15^^,^^16^ We hypothesized that the percutaneous approach would produce greater variability in the location of the screw entry point on the metacarpal head articular surface. This variability, especially more volar starting points, could impact the overall extent of articular surface damage at the MCP joint.

Fixation was performed using Exsomed Innate screws (Acumed), chosen to represent a larger diameter screw. Recent data suggest a 4.0 mm screw is optimal for canal fit in most metacarpals, whereas most prior retrograde fixation studies have used smaller screws (2.5 or 3.0 mm).^9^^,^^11^, ^12^, ^13^ Therefore, examining articular involvement with larger screws allowed assessment of a potentially greater articular defect. Screws of either 3.6 mm or 4.5 mm diameter were selected as appropriate for each metacarpal. All fixation procedures were performed by a fellowship-trained hand surgeon following the Innate surgical technique guide.

### Measurement of articular involvement

After screw placement, the location and percentage of articular surface damage were assessed. For percutaneous approaches, the joint was opened via a paratendinous incision to visualize the articular surface. En face photographs of the metacarpal head were taken and analyzed using ImageJ software (National Institutes of Health) to quantify the articular defect, as described previously.^12^^,^^17^ The screw entry point was measured as a percentage from the dorsal cortex and deviation from the midline. The percentage of the articular surface violated was calculated.

**Figure 3:**
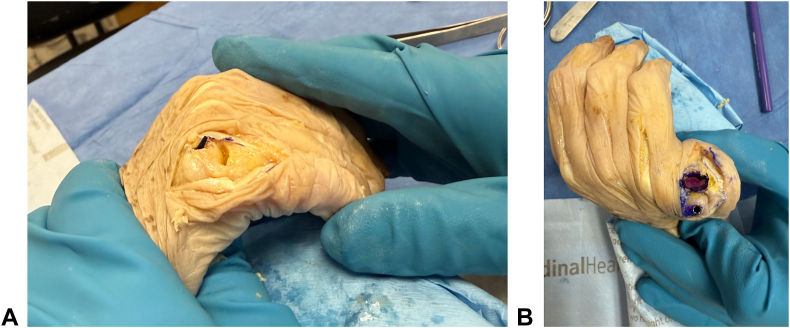
Representative photographs of the marker placement and proximal phalanx articular wear measurements following simulated MCP joint motion. **A** Maximum flexion. **B** Maximum extension of the MCP joint.

To map the articulation of the metacarpal head defect on the proximal phalanx, a small marker was placed 1 mm beyond the metacarpal articular surface. Marker placement ensured dye transfer to the proximal phalanx cartilage simulating contact to the phalanx articular base (Fig. 3). The MCP joint was then moved through full range of motion—full extension to full flexion with radial and ulnar deviation. The percentage of articular surface contacted on the proximal phalanx throughout a full arc of motion was calculated. Of note, the extensor tendon complex was clamped during motion testing to prevent excessive joint movement due to altered mechanics.

### Statistical analysis

The primary outcome was the percentage of the proximal phalanx articular surface contacted by the metacarpal head defect throughout the full arc of MCP joint motion. Secondary outcomes included the location and percentage of articular surface involvement on the metacarpal head. Outcomes were compared between the open paratendinous and percutaneous approaches. Descriptive statistics summarized the findings, and independent *t* tests were used for group comparisons. This study was granted exempt status as it did not involve living human subjects.

## Results

Six fresh-frozen cadaveric specimens (24 metacarpals) were included, consisting of two males and four females (Table 1). The average presurgical MCP joint range of motion was 87° flexion to 42° extension, with no significant differences between specimens. A 4.5 mm Innate screw was used for fixation in all but one metacarpal, where a 3.6 mm screw was used in a female’s fourth metacarpal.

**Table 1:** Characteristics of Cadaveric Specimens Used in the Study

Specimen	Age	Sex	Laterality
1	82	F	R
2	87	F	R
3	63	F	R
4	74	M	L
5	74	M	R
6	82	F	L

The average entry point on the metacarpal articular surface ranged from 37% to 71% measured from the dorsal cortex, and from 12% ulnar to 21% radial relative to the midline. Fractures fixed via the percutaneous approach had significantly more volar starting points and greater variability in the ulnar-radial plane compared with the open paratendinous approach (*P* < .05; Table 2). The percentage of articular surface damage on the metacarpal head caused by screw insertion ranged from 5% to 12%. Larger articular defects were observed with 4.5 mm screws and in smaller metacarpals (middle and ring fingers; *P* < .05).

**Table 2:** Location and Percentage of the Articular Involvement of the Metacarpal Head and Contact Mapping With Proximal Phalanx Articular Surface

Digit	Metacarpal Articular Violation, % (SD)	Radial Ulnar Distance From Center, % (SD)		*P*	Volar Dorsal Distance From Center, % (SD)		*P*	Contact % Articular Proximal Phalanx (%)	
		Open	Perc		Open	Perc		Open	Perc
Index	9.03 (1.63)	1.41 (1.13)	13.66 (4.63)		3.40 (2.46)	12.63 (8.33)		100	100
Middle	6.38 (1.25)	2.27 (2.32)	5.09 (2.97)		7.40 (3.15)	3.95 (1.39)		100	100
Ring	7.57 (2.06)	4.76 (4.44)	11.19 (10.83)		7.29 (5.74)	7.70 (8.67)		100	100
Little	9.12 (2.03)	2.16 (0.80)	3.51 (2.01)		6.68 (5.58)	2.69 (2.08)		100	100
Average	8.03 (2.02)	2.65 (2.58)	8.36 (6.84)	<.05	6.19 (4.18)	6.74 (6.61)	<.05	100	100

In every cadaver, 100% of the proximal phalanx articular surface—including all regions of the base—articulated with and was contacted by the cartilage defect on the metacarpal head throughout the full range of MCP joint motion, including flexion–extension combined with radial and ulnar deviation. This indicates that the articular defect on the metacarpal head engages with the entire opposing surface of the proximal phalanx during normal joint movement. Additionally, the percutaneous technique resulted in more variable screw placement on the metacarpal articular surface, with a significantly higher likelihood of volar and off-center entry points (*P* < .05).

## Discussion

Retrograde intramedullary screw fixation for metacarpal fractures has increased rapidly in global use.^1^, ^2^, ^3^, ^4^, ^5^, ^6^, ^7^ Although it offers advantages over K-wires and plate fixation, concerns persist regarding violation of the extensor mechanism and damage to the MCP joint articular surface, with a potential for long-term arthritis.^7^^,^^10^^,^^13^ Despite multiple clinical studies reporting good-to-excellent short-term results, there have been no long-term studies on the effect of articular or extensor complex damage. A recent systematic review and meta-analysis of all studies published on retrograde metacarpal screw fixation found an average follow-up time of only 9 months (range: 3–19 months) in studies published until 2022.^6^

Proponents contend that arthritis risk is low because the proportion of the metacarpal head articular surface violated by the screw is small and the dorsal entry point may limit defect–proximal phalanx articulation.^11^^,^^12^ Straszewski et al^12^ reported an average of 3.9% articular damage, with screw–proximal phalanx engagement in 70% of specimens, concluding that these features may mitigate joint destruction. However, their cadaveric models had reduced MCP motion (1–85°), smaller screws (2.5–4.0 mm), and possible specimen stiffness—factors that likely underestimated damage. Similarly, ten Berg et al^11^ found 4%–5% damage on CT in vivo using 2.4–3.0 mm screws.

Current fixation principles recommend screws ≥4.0 mm for optimal canal fill and stability, as smaller screws risk displacement from inadequate bone–implant interface.^13^^,^^18^ In our study, 3.6 mm and 4.5 mm screws produced significantly larger articular defects—with an average of 8% of the metacarpal head—exceeding prior reports.

Importantly, we evaluated functional contact between the defect and the proximal phalanx articular surface rather than metacarpal damage alone. This better reflects arthritis risk, as cartilage degeneration depends on mismatch and contact forces during motion. Across all specimens, 100% of the proximal phalanx articular surface contacted the metacarpal defect throughout full MCP flexion–extension with radial/ulnar deviation. More volar and off-center screw entry points, common with the percutaneous approach, may further amplify this wear pattern.

Overall, our findings led us to believe there is potential for disruption of normal joint mechanics due to contact between the metacarpal head defect and the articular surface of the proximal phalanx base. This may lead to long-term cartilage damage and MCP arthritis. If we only focus on the proportion of damaged metacarpal head with retrograde screw instrumentation, this may underestimate a larger influence as the joint undergoes normal motion. With this technique, there have been no long-term outcome studies conducted on the effect of violation of the articular cartilage, and little discussion of complications from similar techniques in orthopedics.^6^^,^^19^ Fairly analogous is intramedullary nailing for tibial shaft fractures, which result in over 70% of patients with lasting pain after tibial nailing and 36% in the long-term develop arthritis without obvious mechanical malalignment.^20^^,^^21^

This study is limited by the number of cadaveric specimens; however, the consistency of results across all samples suggests that this is unlikely to have influenced the overall conclusions. We used larger screws than those reported in prior literature, which produced a larger articular defect; however, this would not be expected to alter the articulation at the tip of the marker in our model. The marking technique allowed for precise visualization of the contact point at the articular surface, and no deviation in this point should be observed despite the increased screw size.

Although retrograde screw fixation offers significant advantages—particularly over K-wire and plate-and-screw fixation—there remains concern regarding long-term MCP joint function and the potential for arthritis. This study builds upon prior work quantifying the insult to the metacarpal head, now demonstrating that the entire articular base of the proximal phalanx articulates with the defect throughout the arc of motion. Although our study cannot establish the direct clinical impact of this finding, it clearly identifies a previously underrecognized contact pattern that warrants further investigation. This observation provides a foundation for future work aimed at determining whether such defects contribute to long-term MCP dysfunction or arthritis.

## Conflicts of Interest

Dr Nicholson is a consultant for Stryker and Meduloc. Dr Hustedt is a consultant for Arthrex, Acumed, and Meduloc. No benefits in any form have been received or will be received by the other authors related directly to this article.
